# Gray Matter Correlates of Creativity in Musical Improvisation

**DOI:** 10.3389/fnhum.2019.00169

**Published:** 2019-05-22

**Authors:** Cameron Arkin, Emily Przysinda, Charles W. Pfeifer, Tima Zeng, Psyche Loui

**Affiliations:** ^1^Department of Psychology, Wesleyan University, Middletown, CT, United States; ^2^Department of Medicine, University of Rochester, Rochester, NY, United States; ^3^Department of Psychology, University of Pennsylvania, Philadelphia, PA, United States; ^4^Department of Music, Northeastern University, Boston, MA, United States; ^5^Department of Psychology, Program in Neuroscience and Behavior, Wesleyan University, Middletown, CT, United States

**Keywords:** creativity, improvisation, gray matter, VBM, music

## Abstract

Creativity has been defined as requiring both novelty and effectiveness, but little is known about how this standard definition applies in music. Here, we present results from a pilot study in which we combine behavioral testing in musical improvisation and structural neuroimaging to relate brain structure to performance in a creative musical improvisation task. Thirty-eight subjects completed a novel improvisation continuation task and underwent T1 MRI. Recorded performances were rated by expert jazz instructors for creativity. Voxel-based morphometric analyses on T1 data showed that creativity ratings were negatively associated with gray matter volume in the right inferior temporal gyrus and bilateral hippocampus. The duration of improvisation training, which was significantly correlated with creativity ratings, was negatively associated with gray matter volume in the rolandic operculum. Together, results show that musical improvisation ability and training are associated with gray matter volume in regions that are previously linked to learning and memory formation, perceptual categorization, and sensory integration. The present study takes a first step towards understanding the neuroanatomical basis of musical creativity by relating creative musical improvisation to individual differences in gray matter structure.

## Introduction

The study of creativity and flow are central to positive psychology and neuroscience. We often know when we are experiencing creative works of art; yet it is impossible to find a single dimension along which to rank all works of creative genius. This is especially true of music. The standard definition of creativity in the psychological literature requires both novelty and effectiveness (Runco and Jaeger, 2012). Despite its clear importance (Guilford, 1950), the topic of creativity remains challenging precisely because novelty, quality, and appropriateness to audience are all difficult to quantify (Baer, 1993).

Efforts to understand creativity have come from multiple methods. Historiometric studies have provided insight into exceptional creativity, also known as “Big C” creativity (Simonton, 2014), and many such studies have focused on composers such as Mozart as paragons of human creativity (Gardner, 1998). Theoretical work posits the cognitive mechanism of “blind variation and selective retention” (Campbell, 1960; Simonton, 2013) as fundamental to the creative process. In this model, information is modified, recombined, and generated pseudorandomly (“blind variation”) to give rise to many possible novel ideas; once the possible ideas are generated, the best ideas are retained (“selective retention”) and refined to become the creative product (Campbell, 1960; Simonton, 2013). To capture this process of “blind variation,” psychometricians have designed divergent thinking tests such as the Torrance Test of Creative Thinking, in which subjects are given short prompts and are expected to generate multiple answers, and subjects’ responses are scored on fluency, originality, flexibility, and elaboration (Torrance, 1968b). While the scoring of fluency can be relatively automatic in divergent thinking tests, scoring of originality by raters can be somewhat subjective; thus consensual assessment between multiple raters is preferred (Amabile, 1982). Another issue concerns the extent to which laboratory tasks such as the Torrance Test of Creative Thinking truly capture the process of creativity in real time, and whether the divergent thinking tests are in fact measuring the same construct as that which was studied historiometrically in great musicians and composers (Baer, 2008).

In music, creativity is seen in composition and in performers’ interpretations, but also especially in improvisation such as in jazz music, which requires generating musical ideas in real time. For this reason, jazz improvisation has long been viewed as a prominent example of improvisational creativity in the Western culture (Sawyer, 1992). In recent years, the science of music has seen a flourishing of studies that take a neuroscientific approach, and the study of jazz improvisation is no exception. Musical improvisation has been studied as a prototypical form of spontaneous creative behavior.

Time-sensitive measures such as electroencephalography and event-related potentials (EEGs and ERPs), have shown both spectral and temporal differences in brain activity among groups of subjects with varying levels of improvisation training (Vuust et al., 2012; Przysinda et al., 2017; Goldman et al., 2018). Results from ERP studies have shown that people with different levels of improvisation training differ in how they react to unexpected musical events in a very time-sensitive manner: while all groups of participants are sensitive to unexpected musical events, trained jazz musicians notice the unexpectedness earlier, as early as 200 ms after event onset, whereas classical musicians continue to show sensitivity well after jazz improvising musicians at 800 ms (Przysinda et al., 2017). Goldman et al. (2018) showed that experienced improvisers responded more quickly to functional deviants (as opposed to exemplar deviants as defined by music-theoretical function), with the differences around 200 ms and 450 ms after chord onset predicting the behavioral advantage of experienced improvisers (Goldman et al., 2018). Furthermore, EEG studies in creativity generally show that alpha-band activity plays a key role in the creative process, with some additional contributions from the theta and gamma bands (Stevens and Zabelina, 2019). Specifically in music, trained improvisers showed higher right frontal alpha band activity, especially during improvisation (Lopata et al., 2017). Additionally, jazz improvising pianists showed more beta power increase whereas classical pianists showed enhanced theta band activity during the perception of syntactically inappropriate or unexpected chords (Bianco et al., 2018).

The first functional Magnetic Resonance Imaging (fMRI) studies compared spontaneous improvisation against controlled performance on the keyboard, and revealed a network of activations and deactivations in medial prefrontal cortex (MPFC) and dorsolateral prefrontal cortex during jazz improvisation (Limb and Braun, 2008). Since then the majority of neuroimaging studies in musical creativity have used functional MRI to relate behavioral performance during improvisation to brain activity (Donnay et al., 2014; Pinho et al., 2014). As jazz improvisation can be thought of as spontaneous generation of auditory-motor sequences (Berkowitz and Ansari, 2008), neuroimaging studies on jazz improvisation can inform the more general neuroscience of motor behavior. As reviewed in recent literature (Beaty, 2015), results have shown differences related to improvisation training in multiple brain regions and at various time-points throughout perceptual and cognitive processing. Many findings centered around the prefrontal cortex, but the precise findings differ among studies. Within the prefrontal cortex, some studies have observed increased activity in the MPFC and downregulation of the lateral prefrontal cortex during improvisation (Limb and Braun, 2008; Liu et al., 2012). In addition to MPFC, other mesial activity has also been observed during improvisation, including the cingulate cortex and supplementary motor area (Bengtsson et al., 2007; Limb and Braun, 2008; de Manzano and Ullén, 2012; McPherson et al., 2016). The SMA and cingulate cortex belong to different resting state networks and are related to transient and sustained cognitive control respectively (Dosenbach et al., 2008; Christoff et al., 2016); both types of cognitive control are likely involved in musical improvisation and are difficult to disentangle in an improvisation task.

Findings also differ between studies depending on the specific cognitive demands during musical improvisation tasks. One study found higher activation in language-related areas in the lateral prefrontal cortex, specifically the pars triangularis and pars operculum of the left inferior frontal gyrus (Broca’s area), especially during socially interactive improvisation (“trading fours”; Donnay et al., 2014). Another study additionally manipulated emotional intent and showed that the valence of the target emotion affected activity as well as functional connectivity in the prefrontal cortex (McPherson et al., 2016). The design of control conditions in these task-based experiments could also explain some differences in results. In functional MRI tasks, the control condition typically also involves the production of auditory-motor sequences, but without spontaneous idea generation: Control tasks have included rote repetition of an overlearned sequence, such as a scale, or a previously memorized melody (Limb and Braun, 2008) or the reproduction of a previously produced performance (Bengtsson et al., 2007). This control task may require more memory, specifically more active maintenance and retrieval strategies, and thus a contrast between improvised and control conditions often reveals deactivations in areas that maintain short-term memory, such as the dorsolateral prefrontal cortex. In addition to these differences in task demands and experiment design, discrepancies necessarily arise in task fMRI studies due to the inherent variability in the mental process of improvisation: at a given moment in the improvisation task, subjects could be utilizing any number of available mental capacities (e.g., visuospatial and/or auditory/phonological components of working memory, autobiographical memory recall, motor planning, attentional selection, and affective communication, just to name a few) to engage in the idea generation and evaluation process. This poses an inherent challenge in task fMRI studies of jazz improvisation (Loui, 2018).

Having identified challenges with task-based neuroimaging data, it becomes clear that a task-independent neuroanatomical comparison could add value to this discussion. Associations between creativity in musical improvisation and individual differences in gray and white matter structure may provide clues as to the fundamental neurobiological underpinnings of musical improvisation ability (Loui, 2018); furthermore, they may offer insights into whether musical improvisation involves shared or distinct networks from non-musical creativity tasks more generally.

Several studies have related neuroanatomical measures to creativity as assessed by non-musical creativity tasks, such as divergent thinking tasks done outside the scanner, and observed associations between creative behavior and regional variations in the posterior cingulate cortex, the lingual gyrus, the angular gyrus, and the orbitofrontal cortex (Jung et al., 2010). Self-report measures of creativity, as measured by the Creativity Achievement Questionnaire (Carson et al., 2005), are inversely correlated with gray matter volume in the cingulate cortex and SMA (Chen et al., 2014), but performance on a divergent thinking task showed positive associations with gray matter volume in the caudate, precuneus, midbrain, and middle frontal gyrus (Takeuchi et al., 2010). More recently, Shi et al. (2017) directly compared gray matter volume between self-report measures of artistic and scientific creativity, and found that artistic creativity was negatively associated with gray matter volume in the SMA and cingulate cortex whereas scientific creativity was positively correlated with the gray matter volume in more lateral prefrontal structures, specifically the left middle frontal gyrus and left inferior occipital cortex. These findings suggest that creativity may be supported by multiple distributed regions in a domain-specific, rather than a domain-general manner. Thus, studies that use domain-specific measures of creativity may add value to our understanding of creativity as a construct.

In musical creativity specifically, a recent study has related cortical surface area and volume from structural MRI data to musical creativity (Bashwiner et al., 2016). They found that cortical surface area in superior frontal gyrus, left planum temporale, and right middle temporal gyrus, and subcortical volume in left amygdala, were correlated with musical creativity ratings. These findings were important as they were first to relate musical creativity to brain structure; however, creativity was assessed by self-report measures and not independently verified. Combining a behavioral task of musical improvisation with MRI measures of individual differences in brain structure will yield direct associations between improvisation ability and brain structure, thus shedding light on the neural correlates of real-time creative behavior while circumventing methodological challenges as reviewed above. Furthermore although these measures of brain structure are task-invariant (i.e., not dependent on task-induced activations), they may change over time as a result of training-induced plasticity even in adults, as shown by numerous studies in the neuroanatomical changes as a function of musical training independent of creativity (Schlaug, 2001, 2015; Pantev et al., 2003, 2009; Bengtsson et al., 2005; Imfeld et al., 2009; Halwani et al., 2011; Elmer et al., 2012, 2013, 2016; Herholz and Zatorre, 2012; Oechslin et al., 2013, 2018; Gärtner et al., 2013; Kleber et al., 2016; Karpati et al., 2017; Moore et al., 2017; Li et al., 2018). Thus, a better understanding of neuroanatomical correlates of musical creativity may enable future interventions and training programs that specifically target the plasticity of these neuroanatomical regions with the goal of promoting musical creativity.

Here, we report the first results from a combined behavioral and neuroimaging study to test the hypothesis that individual differences in creative musical behavior are associated with differences in gray matter structure. We use a behavioral task of musical improvisation to assess real-time creativity in individuals with a broad range of musical training, similar to recent studies from a developmental perspective (Ilari et al., 2017). Expert ratings of musical creativity are used as predictors in a voxel-based morphometry (VBM) study to relate gray matter structure to musical improvisation behavior.

## Materials and Methods

### Subjects

Thirty-eight subjects from Wesleyan University and the Hartt School of Music participated in return for financial compensation or course credit. All subjects gave written informed consent as approved by the Institutional Review Boards of Wesleyan University and the Hartford Hospital. They then completed a survey of their background including demographic information, information on their age of onset and duration of general musical training and training in jazz and in musical improvisation, and self-rating of skills in improvisation. Care was taken to recruit a racially/ethnically diverse and gender-balanced sample that was representative of the student population. While the subjects were racially/ethnically diverse, there was a skew towards male subjects. To control for possible confounds of gender in our results, we incorporate gender as a covariate in all of our analyses. Subjects also completed background tests as part of prescreening procedures for our study. These included the non-verbal subtest of the Shipley Institute of Living, which is a correlate of IQ that is used to rule out intellectual impairment (Shipley, 1940), digit span for short term memory (Baddeley, 2003), and a pitch discrimination threshold-finding task (Loui et al., 2008) to screen for tone-deafness. Table 1 shows demographics, music training variables, and results on baseline tests.

**Table 1:** Subject demographics and musical training information.

	Mean ± SD
Gender	Female *n* = 11; Male *n* = 27
Age	21.87 ± 3.02
Handedness	Left *n* = 4; Right *n* = 34
Ethnicity/Race	Asian and Pacific Islander *n* = 6, Asian/Caucasian *n* = 2,
	Black/African American *n* = 4, Hispanic/Latino *n* = 3, White/Caucasian *n* = 23
Pitch Discrimination Perception (Hz)	6.92 ± 7.94
Digit Span (digits)	7.55 ± 1.61
Shipley (raw score)	17.14 ± 1.73
Age of onset of musical training (years)	8.31 ± 2.56
Duration of musical training (years)	7.36 ± 4.10
Duration of improvisation training (years)	3.59 ± 3.73
Musical instruments played	piano (16), guitar (12), drums (5), clarinet (5), bass (5), violin (4), saxophone (4), voice (3), flute (2), pipa (1), trumpet (1)

### Stimuli

**Figure 1:**
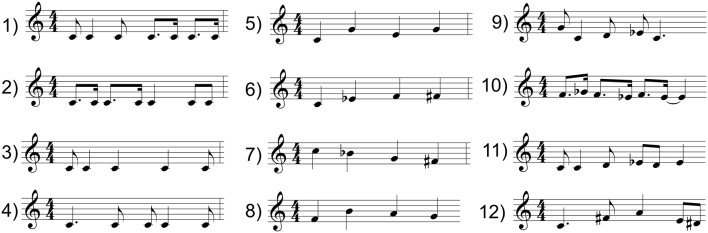
Musical prompt for the improvisation continuation task.

Twelve musical prompts were composed for this experiment. Each prompt lasted 2.4 s (1 measure = 4 beats at 100 bpm). The prompts varied in either rhythm or pitch. Figure 1 shows the 12 musical prompts, and example recordings of the prompts are also available online here: https://doi.org/10.6084/m9.figshare.6590489.v1. All prompts were presented in MIDI grand piano timbre throughout the experiment.

### Procedures

#### Improvisation Continuation Task

Twelve different trials were presented from a computer. Each trial consisted of a listening phase (two measures), a continuation phase (eight measures), and an improvisation phase (eight measures). Visual cues were given on the screen throughout the listening phase (“Listen”), the continuation phase (“Play along”), and the improvisation phase (“Improvise”). In addition, a metronome was presented at 100 bpm to keep time throughout the entire trial.

Subjects were instructed to listen to the clip two times during the listening phase, to play along with the prompt eight times during the continuation phase, and then to improvise in the most creative way they could be based on the prompt given, for another eight measures until the metronome stops. No additional instructions were given on how to improvise, nor were any guidelines given for what constitutes creativity. All subjects completed this task on a Casio PX 150 MIDI keyboard. Subjects who self-identified as playing other instruments additionally performed on their instrument of choice. MIDI data were collected through Max/MSP using the borax function, which recorded the pitch, velocity, duration, and inter-onset interval for each note received by the keyboard. All performances were also recorded using a Zoom Q8 video camera.

#### MRI

T1 images were acquired from all participants in a 3T Siemens Skyra MRI scanner at the Olin Neuropsychiatry Research Center at the Institute of Living. Anatomical images were collected using a T1-weighted, 3D, magnetization-prepared, rapid-acquisition, gradient echo (MPRAGE) volume acquisition (Axial acquisition, 224 slices, FOV = 256 mm, TE = 2.09 ms, TR = 2,400 ms, flip angle = 8°, voxel resolution = 0.8 × 0.8 × 0.8 mm^3^). We also acquired resting state fMRI and DTI images, which will be described in a separate report.

### Data Analysis

#### Improvisation Continuation Task Analysis

Example audio files of subjects’ production are available online at https://doi.org/10.6084/m9.figshare.6590489.v1. Audio was extracted from video recordings and rated for creativity by two professional jazz instructors (Rater 1 and Rater 3) and one experienced jazz improvising musician (Rater 2). Expert raters were asked to listen to each anonymized recording of each improvisation, and to rate the recording on a scale of 1–6 for creativity and imagination, with 1 being “Not creative and/or imaginative” to 6 being “Creative and/or imaginative.” Rater 1 completed the full dataset of 456 ratings (38 subjects * 12 trials each). Rater 2 completed ratings for 10 subjects (12 trials each * 10 subjects = 120 ratings), but stopped after 120 ratings due to lack of interest. Rater 3 completed 432 ratings (36 subjects * 12 trials each); two subjects were tested after Rater 3 already completed the other ratings; thus the last two subjects were not rated by Rater 3. Each rater’s ratings were averaged across the 12 trials performed by each subject to obtain an average score given by each rater to each subject. We then obtained an averaged creativity rating for each subject by averaging across all available raters’ data for that subject.

#### MRI Analysis

VBM analysis (Ashburner and Friston, 2000) was performed on T1 images to relate gray matter variations to behavioral measures. First, we extracted T1 images of the brain from non-brain voxels using the Brain Extraction Tool (BET) in FSL (Jenkinson et al., 2012). Brain masks were checked to ensure that they accurately covered the entire brain. Then in SPM12, the images were realigned by setting the origin to the anterior commissure. Using the VBM toolbox in SPM12, the brain-extracted images were normalized relative to the canonical image (avg152T1.nii). Images were then segmented into gray matter, white matter, and cerebrospinal fluid. The resulting gray matter images were smoothed using a 12 mm Gaussian kernel. Multiple regressions were run on the behavioral dependent variable of averaged creativity score, with gender and overall brain volume added as covariates of no interest. A whole-brain regression was not significant at the *p* < 0.05 FWE-corrected or FDR-corrected levels. We, therefore, applied a combined threshold with voxel-wise significance level of *p* < 0.001 (uncorrected) and a cluster-correction of *k* > 10 voxels, to capture results that surpass both a voxel-wise and an extent threshold. While the use of uncorrected voxel-wise significance level was relatively liberal, the additional application of the extent threshold served to reduce the likelihood of type 1 errors.

## Results

### Behavioral Results

Mean creativity rating across the three expert raters was 3.19 (SD = 0.96). Cronbach’s Alpha across the three raters was 0.932, confirming high inter-rater reliability. Cronbach’s alpha was calculated in SPSS using list-wise deletion to account for missing data resulting from incomplete datasets from raters. Since list-wise deletion does not take into account the subjects that only received two out of three possible ratings, we also separately show the pairwise correlation matrix between each pair of raters. The three raters’ average scores were all highly correlated, as shown in the inter-rater correlation matrix in Table 2. The available ratings for each subject were averaged across the raters to derive an averaged creativity ratings score, ranging from 1 (Not creative and/or imaginative) to 6 being (Creative and/or imaginative). The resulting averaged creativity score was normally distributed (Kolmogorov-Smirnov test: *D*_(38)_ = 0.9, n.s.).

**Table 2:** Inter-rater correlation matrix showing correlation coefficients (*r*) for each pair of raters.

	Rater 1	Rater 2
Rater 2	0.931	
Rater 3	0.860	0.804

Multiple regression was used to test if training and performance on background tests predicted creativity. We ran a multiple linear regression analysis on the dependent variable of averaged expert ratings of creativity, with the independent variables of duration of improvisation training and duration of general musical training, as well as scores on pitch discrimination, digit span, and Shipley tests. This regression was significant, with the combined factors explaining 69% of the variance (*R*^2^ = 0.693, *p* = 0.003). Duration of improvisational training was the most significant predictor of creativity ratings (*β* = 0.75, *p* < 0.001), whereas duration of general musical training did not significantly predict creativity ratings (*β* = 0.13, *p* = 0.37). Performance on pitch discrimination (*β* = −0.212, *p* = 0.156), Digit span (*β* = −0.251, *p* = 0.128), and Shipley (*β* = 0.112, *p* = 0.46) baseline tests did not significantly predict creativity ratings.

### Voxel-Based Morphometry

**Figure 2:**
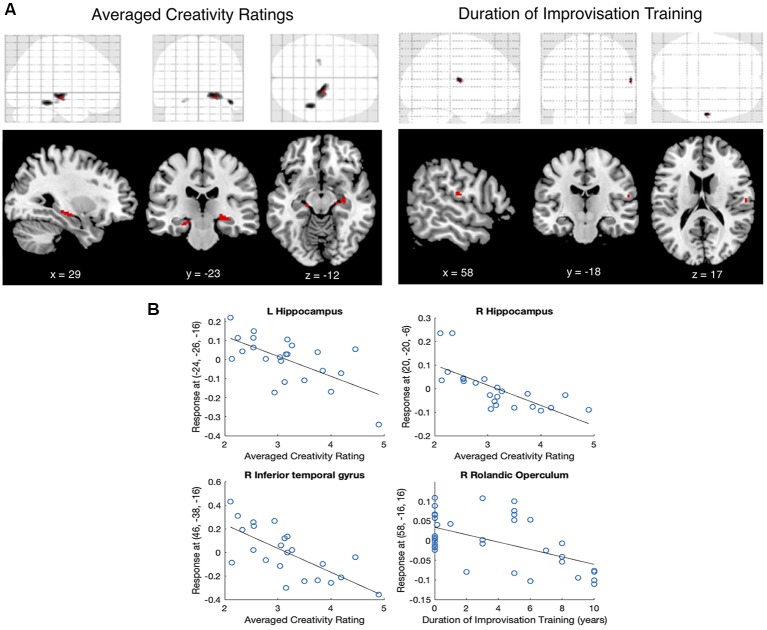
**(A)** Voxel-based morphometry (VBM) regression on averaged creativity ratings and the duration of improvisation training (*T* > 3.53, *p* < 0.001, *k* > 10). Top row shows glass-brain representations of the whole-brain regressions; Bottom row shows significant clusters overlaid on a T1 template. **(B)** Scatterplots showing relationships between averaged creativity ratings and gray matter signal at left and right hippocampus, between averaged creativity ratings and right inferior temporal gyrus, and between the duration of improvisation training and the right rolandic operculum.

A whole-brain regression on gray matter volume with the covariate of averaged creativity ratings showed no significant voxels at the *p* < 0.05 FWE- or FDR-corrected levels. However, there were three significant clusters that surpassed the combined peak and cluster thresholds (*T* = 3.53, *p* < 0.001 uncorrected, extent threshold *k* = 10 voxels). These clusters were identified using the Automated Anatomical Labeling (AAL) atlas (Tzourio-Mazoyer et al., 2002) as being in the left and right hippocampus and the right inferior temporal gyrus. Scatterplots showed a negative relationship between averaged creativity ratings and gray matter signal in all three regions: participants whose production was rated as more creative had lower gray matter signal in all three regions (Figure 2B).

A whole-brain regression on gray matter volume with the covariate of duration of improvisation training showed a single cluster that surpassed both *t* and *k* thresholds, in the right rolandic operculum. Scatterplots again showed a negative relationship between the duration of improvisation training and gray matter signal: participants who had more improvisation training had lower gray matter signal in the rolandic operculum. Figure 2 shows anatomical locations and sizes of these clusters (Figure 2A), and their correlations with averaged creativity ratings and with improvisation duration (Figure 2B). Table 3 shows the clusters in MNI coordinates.

**Table 3:** Significant clusters (MNI coordinates) from whole-brain regression analysis of variations in gray matter volume with creativity ratings.

Contrast	Region	Hemisphere	T	*k*	*x*	*y*	*z*
Creativity	Hippocampus	R	4.63	179	20	−20	−6
	Inferior temporal gyrus	R	4.58	76	46	−38	−16
	Hippocampus	L	3.84	22	−24	−26	−16
Duration of Improv Training	Rolandic Operculum	R	3.77	15	58	−16	16

## Discussion

In this study, we combine behavioral and voxel-based morphometry methods to relate brain structure to creativity in musical improvisation. Results show that expert ratings of creativity on an improvisation task are associated with differences in gray matter structure and that these associations are distinct from neuroanatomical correlates of training in musical improvisation.

Our musical improvisation continuation task is modeled after previous studies and is designed to be doable by participants of all levels of training (Pinho et al., 2014; Ilari et al., 2017). Like all creative tasks, there is no unique correct answer for each trial, but some utterances are clearly more creative than others. In that sense, our task parallels the more domain-general divergent thinking task (Torrance, 1968a). To quantify creativity in these situations, the most common approach is to rely on expert ratings, such as by professional jazz musicians. Here, we show that there is consistent agreement between the expert ratings of musical improvisations, suggesting that musical creativity can be reliably assessed and compared between participants in a behavioral paradigm.

Individual differences in gray matter volume as identified in voxel-based morphometry may reflect variations in neuronal cell bodies themselves and/or from differences in relative density of cell bodies which gives rise to gyrification patterns, which may, in turn, facilitate connectivity towards those cell bodies (Ashburner and Friston, 2000). Here, we find that creativity ratings are negatively associated with gray matter volume in the bilateral hippocampus and the right inferior temporal gyrus. These clusters show no overlap with the cluster that is negatively associated with duration of improvisation training, which is in the rolandic operculum. The consistent patterns of negative correlations between gray matter volume and variables of interest have been observed in other studies: as reviewed in the introduction, measures of creative achievement were inversely correlated with gray matter volume in the anterior cingulate and the SMA (Chen et al., 2014; Shi et al., 2017). In a previous study relating gray matter volume to rhythm perception and production, temporal discrimination abilities were found to be inversely correlated with gray matter volume in the cerebellum (Paquette et al., 2017). As voxel-based morphometry is a voxel-wise comparison of the local concentration of gray matter between individuals (Ashburner and Friston, 2000), the results are agnostic to the direction of the difference between participants. Lower gray matter volume in more creative and more highly trained musical improvisers may be due to more densely packed neuronal cell bodies, more neuronal and/or synaptic pruning, and/or differences in the distribution of glial cells leading to a less defined gray-white matter boundary in the significant regions among successful improvisers. While at present we cannot disentangle these possible interpretations, in future studies we plan to use cortical thickness and surface area, subcortical volume, white matter volume, and other methods to tease apart these distinct biological mechanisms in a larger sample.

Creativity ratings were negatively associated with gray matter volume in the hippocampus. The hippocampus is primarily involved in learning and memory formation; recent work has linked this structure to creativity tasks as well. A recent voxel-based morphometry study found that hippocampal volume was correlated with performance on the remote associate’s test, a verbal creativity task (Tu et al., 2017). Among amateur and expert musicians, previous work has shown that hippocampal volume is significantly correlated with fluid intelligence as assessed by the Raven’s Advanced Progressive Matrices (Oechslin et al., 2013). A jazz guitarist whose left hippocampus was surgically removed due to an arteriovenous malformation, lost his musical capabilities while acquiring profound retrograde amnesia following the surgery. However, through long-term training and associations, he was able to recover completely in his ability to improvise music, despite chronic impairment in verbal memory but not visual memory tasks (Galarza et al., 2014). This suggests that temporal lobe in both hemispheres, including both hippocampi, are involved in musical improvisation, but also that improvisation ability can be recovered even with only one intact hippocampus (Duffau, 2014). Here, the hippocampal clusters that are inversely correlated with creativity ratings span the middle to anterior hippocampus and parahippocampal gyrus in the right hemisphere, and the middle to posterior hippocampus in the left hemisphere. The slight asymmetry between left and right hippocampal findings may highlight known dissociations along the anterior-posterior axis of the hippocampus (Poppenk and Moscovitch, 2011). The anterior hippocampus is more associated with perceptual novelty, imagery, and episodic memory formation (Zeidman and Maguire, 2016), whereas the posterior hippocampus is more associated with indexing familiarity to behaviorally relevant stimuli (Strange et al., 1999). As musical improvisation requires sensitivity to perceptual novelty (Przysinda et al., 2017) as well as familiarity with known repertoire (Pressing, 1998), the functions of both anterior and posterior hippocampus are likely at work in musical improvisation.

The inferior temporal gyrus is part of the “what” pathway in the visual system. Specifically, the cluster that we find to be associated with averaged creativity ratings falls in the gray-white matter boundary between the right posterior inferior temporal gyrus and the fusiform gyrus, in an area that is activated during studies that involve categorization of meaningful stimuli ranging from objects such as tools and chairs (Ishai et al., 1999; Creem-Regehr and Lee, 2005; Rice et al., 2014) to bodies (Downing et al., 2001; Peelen and Downing, 2005) and emotional categories in sign language (Emmorey and McCullough, 2009). These categories, although seemingly disparate, may share the characteristic of having action-related properties, in that they are categories of objects and concepts that can afford action (Mahon et al., 2007). Given the role of right inferior temporal gyrus in access to categories, its association with creativity in musical improvisation likely reflects better access to the relevant action-related categorical information (e.g., notes, chords, melodies) among better performers.

The rolandic operculum is anatomically between the parietal and temporal lobes and includes multisensory integration areas including secondary somatosensory cortex. Activity in the rolandic operculum has been reliably observed during interoception which is the awareness of one’s own bodily sensations, such as the awareness of one’s own heartbeat (Blefari et al., 2017). In music, activity in the rolandic operculum has been observed during musical improvisation when emotional improvisation is contrasted against simply improvising with pitch (Pinho et al., 2016). Here, we find that people with more improvisation training show lower gray matter volume in the rolandic operculum. In light of findings that the rolandic operculum is important for the sensory integration of interoceptive signals especially during the production of emotional sounds, our interpretation is that this sensory-interoceptive-emotional integration is especially relevant during improvisation training. This is consistent with known educational strategies in teaching jazz improvisation, which emphasize awareness and anticipation of sensory feedback and simultaneously communicating emotions while maintaining flow (Biasutti, 2015).

Surprisingly, our study found no significant associations with creativity ratings or duration of improvisation training in the prefrontal cortex nor in the frontal lobe. This stands in contrast to two VBM studies on creativity outside of music, which showed negative associations between creativity, in particular, artistic creativity, and gray matter volume in the anterior cingulate cortex and the SMA (Chen et al., 2014; Shi et al., 2017). These differences may be explained by our different approaches in that previous studies used the Creativity Achievement Questionnaire (Carson et al., 2005), a self-report measure that asked questions about successes in various creative domains including music but not specific to improvisation, whereas we incorporated improvisation as the primary behavioral measure in our study. VBM associations with real-time creative ability, as assessed in a musical improvisation task, may tap first and foremost into neural substrates for memory, learning, and categorization, rather than neural substrates for sustained and transient cognitive control as may be required for more long-term production of creative products such as musical composition.

Summarizing the VBM findings, gray matter volume in the bilateral hippocampus and the right inferior temporal gyrus, which are involved in learning, memory formation, and object categorization, together reflect differences in musical creativity as assessed by expert ratings on an improvisation task. Performance on the musical improvisation task shows different neuroanatomical correlates from the duration of musical improvisation training, which is negatively correlated with gray matter volume in the rolandic operculum, a region likely involved in sensory integration.

### Limitations

This study is first to relate musical improvisation behavior to gray matter correlates in brain structure; however, there are several limitations. First, the sample size is relatively small for a VBM study. This could contribute to the major caveat: that our results do not survive correction for multiple comparisons at the *p* < 0.05 FWE or FDR-corrected levels. Second is that the musician participants in our study are relatively early in their musical careers; thus the relationship between creativity and gray matter may not necessarily extend towards more experienced players. Third, more than two-thirds of the participants in this sample are male; this is similar with other neuroimaging studies on musical improvisation (Limb and Braun, 2008; Liu et al., 2012; Donnay et al., 2014; Pinho et al., 2016) and reflects the higher availability of male participants with improvisation training. Studies on the neural correlates of creativity have shown some evidence for gender asymmetry (Ryman et al., 2014); while our sample is too small to separately relate creativity to male and female brain structure, ongoing efforts are focused on recruiting more females to participate in the study. Finally, the current study focuses on relating performance on the improvisation task to gray matter structure of the brain; other work on white matter is ongoing (Zeng et al., 2018), as is additional work on resting state functional connectivity using resting state functional MRI (Belden et al., 2018). Together, the functional and structural (gray and white matter) differences can contribute to our thinking about structural and functional brain networks that enable creativity, and may possibly serve as targets for future training and intervention.

## Conclusion

Taken together, we present first results relating individual differences in brain structure to expert ratings of performance in a musical improvisation task. The results show that individual differences in musical creativity, as assessed by an improvisation task, are associated with regions implicated in memory formation and categorical representation, whereas regions implicated in sensory integration are associated with duration of improvisation training. The use of VBM enables the identification of specific regions in the brain that has demonstrated functions, and knowledge of these functions then shed light on how the brain accomplishes the complex creative task of musical improvisation. Future work will expand the current approach to a larger sample size, and also apply other data-driven measures to predict creativity and relate them to brain structure and function. By relating brain structure to the perceived creativity of musical output, the present preliminary results set the stage for further research towards the neural correlates of musical creativity.

## Ethics Statement

This study was carried out in accordance with the recommendations of “IRBs of Wesleyan University and Hartford Hospital” with written informed consent from all subjects. All subjects gave written informed consent in accordance with the Declaration of Helsinki. The protocol was approved by Wesleyan University and the Hartford Hospital.

## Author Contributions

PL conceptualized the idea behind this manuscript, performed data analyses, and wrote the first draft. CA, EP and TZ acquired and preprocessed the behavioral and neuroimaging data. CA and CP analyzed the behavioral and neuroimaging data. All authors revised the manuscript and approved the submission.

## Conflict of Interest Statement

The authors declare that the research was conducted in the absence of any commercial or financial relationships that could be construed as a potential conflict of interest.
